# What Is “Cold” and What Is “Hot” in Mucosal Ablation for Barrett’s Oesophagus-Related Dysplasia: A Practical Guide

**DOI:** 10.3390/life13041023

**Published:** 2023-04-15

**Authors:** Marco Spadaccini, Ludovico Alfarone, Viveksandeep Thoguluva Chandrasekar, Roberta Maselli, Antonio Capogreco, Gianluca Franchellucci, Davide Massimi, Alessandro Fugazza, Matteo Colombo, Silvia Carrara, Antonio Facciorusso, Pradeep Bhandari, Prateek Sharma, Cesare Hassan, Alessandro Repici

**Affiliations:** 1 Department of Biomedical Sciences, Humanitas University, 20089 Rozzano, Italy; 2 Digestive Endoscopy Unit, Department of Endoscopy, Humanitas Research Hospital, IRCCS, Via Manzoni 56, 20089 Rozzano, Italy; 3 Department of Gastroenterology and Hepatology, Augusta University Medical Center, Augusta, GA 30912, USA; 4 Gastroenterology Unit, Department of Surgical and Medical Sciences, University of Foggia, 71122 Foggia, Italy; 5 Department of Gastroenterology, Portsmouth Hospitals University NHS Trust, Portsmouth PO6 3LY, UK; 6 Department of Gastroenterology and Hepatology, Kansas City VA Medical Center, Kansas City, MO 66045, USA

**Keywords:** Barrett oesophagus, radiofrequency ablation, oesophageal adenocarcinoma, APC, cryotherapy, low-grade dysplasia, high-grade dysplasia

## Abstract

Over the last two decades, endoscopic eradication therapy has been established as the therapeutic strategy of choice for patients with Barrett’s oesophagus-related dysplasia and early oesophageal adenocarcinoma. With a multimodal approach, ablative therapies have been highly effective in achieving remarkable eradication rates of metaplastic epithelium with an acceptable adverse event rate. Among ablative techniques, radiofrequency ablation is currently considered as the first-line option as its efficacy and safety are strongly supported by relevant data. Nevertheless, radiofrequency ablation is costly, and not universally available, or applicable to every situation. Moreover, primary failure and recurrence rates are not negligible. In the last few years, cryotherapy techniques and hybrid argon plasma coagulation have been increasingly assessed as potential novel ablative therapies. Preliminary data have been promising and suggest that they may even have a role as first-line options, alternatively to radiofrequency ablation. The aim of this review is to provide a practical guide for the ablation of Barrett’s oesophagus, with emphasis on the different ablative options.

## 1. Introduction

Barrett oesophagus (BE) is diagnosed when salmon-coloured epithelium replaces the normal stratified squamous epithelium of the oesophagus, and extends for at least 1 cm above the gastroesophageal junction (GEJ) with confirmed intestinal metaplasia (IM) on histopathological examination [1]. BE is caused by chronic mucosal injury due to gastroesophageal reflux disease (GERD), with a prevalence of 1–2% in the general population and 5–15% in GERD population [2,3].

Notably, BE is recognized as the main precursor of oesophageal adenocarcinoma (EAC) [4]. The risk of progression to cancer increases with worsening dysplastic changes in BE. While there is not an augmented risk in BE patients without dysplasia, a higher chance of progressing to EAC has been well-established in those with BE-related dysplasia. Patients with low-grade dysplasia (LGD) and high-grade dysplasia (HGD) have an annual risk of progression to EAC of 0.5% and 7%, respectively [5,6].

Over the last two decades, endoscopic eradication therapy (EET) has dramatically revolutionized the management of BE-related dysplasia and early EAC by decreasing costs, morbidity and mortality, without an inferior efficacy in comparison with esophagectomy, which was the previous standard of care [7,8,9].

EET is a multimodal approach consisting of resection techniques and ablative therapies. Resection techniques are used for the removal of visible lesions with suspected or known dysplasia/early EAC along the BE segment. Ablative therapies are performed after resection of visible lesions with confirmed dysplasia/early EAC or flat dysplastic segments without visible lesions. They destroy all the residual metaplastic epithelium over multiple sessions [10]. Ablation has been shown to be an effective treatment, with the aim of complete eradication of dysplasia (CE-D) and intestinal metaplasia (CE-IM), thus decreasing the progression rate from BE-dysplasia to cancer [11].

Particularly, radiofrequency ablation (RFA) is currently regarded as the first-line ablation technique, due to its good efficacy and safety profile supported by high-quality evidence, outperforming photodynamic therapy (PDT) and argon plasma coagulation (APC), which were more commonly used in the past [12]. In the last few years, novel options, including hybrid APC and cryotherapy techniques, have emerged as valuable options, even potentially challenging the role of RFA as the gold standard option [13,14,15].

In this review, we aim to provide a practical guide for the ablation of BE dysplasia, focusing on the different ablative techniques, trying to underline the most suitable tool for each situation and point out the future evolution and research landscapes.

## 2. Practical Considerations and Concept of Ablation

Prior to ablation, it is crucial that an accurate endoscopic pretreatment assessment is performed using an optical zoom scope with a cap attached to the distal tip to enhance visualization [16]. High-definition white-light examination and virtual and dye-based (acetic-acid) chromoendoscopy evaluation are recommended to evaluate for strictures, ulcerations, scarring from previous treatments, and, most importantly, visible lesions or nodularity [17]. It is mandatory to report the circumferential (C) segment and maximal (M) extent of BE according to the Prague classification [18]. Ablation should only be performed in the presence of flat BE without visible lesions or signs of inflammation (erosions and ulcers), which represent contraindications to ablative therapy. Indeed, as part of EET, visible lesions should be always resected before ablative therapy to avoid the risk of buried BE post ablation. The histological analysis of provided specimens can be fundamental for subsequent therapeutic choices. The dilation of oesophageal strictures should be performed at least 2–3 weeks prior to ablative therapy [7,10,19]. Other contraindications include oesophageal varices due to the risk of bleeding, and prior radiation therapy due to the augmented risk of stricture formation.

The goal of ablative therapy in BE is to achieve CE-D and CE-IM by inducing necrosis of diseased epithelium through thermal, photochemical or freezing damage. The main principle of ablative therapy is that the ablated tissue is then replaced by normal regenerating oesophageal squamous epithelium. Although this concept was first demonstrated more than 20 years ago, the precise underlying mechanism is currently unknown [20]. Surrounding squamous cells and progenitor cells are believed to promote the regeneration of squamous epithelium [21]. Through multiple sessions, usually about every 2–3 months, ablation should be applied to all metaplastic epithelium starting from 5–10 mm distal to the GEJ until there is no more macroscopic and microscopic detection of BE [7,19].

## 3. Indications

EET has been established in the last 20 years as the gold standard therapeutic option for BE-HGD and intramucosal carcinoma (IMC; T1a) [7]. Esophagectomy has a high risk of surgery-related morbidity and mortality [9]. Although no randomized control trial comparing EET with surgery has been conducted, a meta-analysis enrolling more than 800 patients with BE-HGD had a better safety profile (RR 0.38; 95% CI, 0.20–0.73) than esophagectomy with comparable overall remission rate (RR 0.96; 95% CI 0.91–1.01), neoplasia related mortality (relative difference 0; 95% CI 0.02–0.01) and overall survival at 5 years (RR 1.00; 95% CI, 0.93–1.06) [8]. EET has been shown as highly effective and durable for BE with HGD, achieving excellent long-term CE-D and CE-IM rates (>90%) [22,23,24].

The management of BE-LGD is a much-debated topic. In the past, the preferred approach after detection of BE with LGD was surveillance at 6 months and then at 12 months, if LGD was confirmed by a second pathologist with expertise in BE. For the first time, in 2009, a clinical trial randomized patient with LGD to RFA versus sham ablation was conducted. This study showed that patients treated with RFA had a significantly lower risk of developing HGD at 1 year in comparison to those who underwent sham ablation (5% vs. 14%) [22]. A recent meta-analysis of randomized controlled trials showed RFA achieved significantly higher CE-D and CE-IM rates and remarkably decreased the progression of BE-LGD to BE-HGD compared with surveillance (RR, 0.25; 95% CI, 0.07–0.71; *p* = 0.01. Although RFA led to a lower risk of progression to EAC than control, this finding was not statistically significant (RR, 0.56; 95% CI, 0.05–6.76, *p* = 0.65). Additionally, the complications rate after RFA was higher [25]. 

Thus, the updated guidelines consider both EET and close surveillance (every 6 months for the first year and annually thereafter) as viable options and the risks and benefits of both these approaches should be discussed with patients [7,19]. Despite the well-defined annual risk of about 0.5% of developing EAC from LGD, the efficacy data of EET versus surveillance in decreasing the risk of progression to EAC only has moderate level of evidence. Furthermore, other factors have hampered the development of clear indications for EET or surveillance in BE-LGD. Firstly, the diagnosis of LGD is challenging with high interobserver variability among pathologists. Additionally, LGD diagnosed with biopsies can often regress at subsequent endoscopic follow-up biopsies. A multicentre randomized controlled trial is currently ongoing to compare EET and surveillance for management of BE-LGD with endpoints of neoplastic progression [26].

Finally, although some trials evaluating ablative therapy for non-dysplastic BE (NDBE) found -to-excellent CE-IM rates at mid- and long-term follow-up [27,28], the current guidelines do not recommend ablation for patients with NDBE [7,10,19] given the very low risk of progression to EAC [29]. Indeed, invasiveness, costs, potential adverse events, inability to achieve a 100% CE-IM rate and need for post-ablation surveillance hinder the use of pre-emptive ablation in NDBE patients. Nevertheless, some authors believe that EET could be reasonable in specific situations, such as a young male patient with long-segment BE and a family history of EAC, since these features have been recognized as predictive factors of progression to dysplasia and EAC [30]. However, the lack of data prevents a conclusive recommendation on this topic.

## 4. Ablation Techniques

The most commonly used ablative therapies for BE include thermal options, encompassing radiofrequency ablation (RFA), argon plasma coagulation (APC) and hybrid APC, and nonthermal options, such as cryoablation.

### 4.1. Radiofrequency Ablation

RFA uses heat generated by radiofrequency energy to destroy metaplastic tissue, delivering a reliable and uniform depth of penetration through the diseased oesophageal mucosa, which is in turn replaced with neo-squamous epithelium. 

Firstly, oesophageal mucosa must be irrigated and cleaned with N-acetyl cysteine through the water jet channel of the endoscope to remove secretions that could impair the delivery of radiofrequency. Accurate identification of the GEJ and the characteristics of BE segment is required to ensure RFA is delivered safely and only to the desired mucosa. Then, the endoscopist chooses the right RFA catheter according to the morphology of the BE segment, availability, and operator skill set. The most currently used ablation device system is the Barrx FLEX system, which encompasses circumferential devices for circumferential BE segments > 3 cm in length, and focal ablation devices for circumferential BE segments < 3 cm in length or non-circumferential BE [31] (Table 1). 

**Figure 1:**
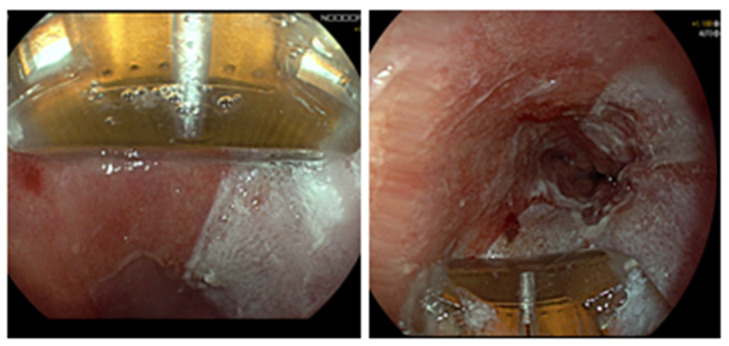
Focal radiofrequency ablation applied through Barrx 90 RFA Focal Catheter.

Focal ablation (usually at 15 J/cm^2^) is used for short segments or islands, either as an initial therapy or during follow-up after circumferential RFA. Focal ablation devices, either those that are attached to the tip of the endoscope or those which are through the scope, are advanced with the endoscope to the target BE segment. Once the ablative surface of the device is positioned against the target BE area under direct endoscopic control, RFA is performed twice. Following ablation, the ablated mucosa with the coagulum is scraped off with the cap mounted on the endoscope and the focal ablation device is removed and cleaned. Then, the process is repeated and RFA is reapplied again (Figure 1) [31].

Of note, the technique described above is the most widely used RFA protocol, which is regarded as the “standard protocol”. It encompasses a cleaning phase, where the ablated mucosa (after one application at 12 J/cm^2^ for circumferential RFA or two applications at 15 J/cm^2^ for focal RFA) is scraped and the ablation devices are removed and cleaned before the second RFA round. Despite its efficacy, this protocol is lengthy and requires many intubations. To overcome these limitations, a “simplified” protocol without the cleaning phase between RFA rounds has been developed. Two randomized trials by van Vilsteren et al. showed standard and simplified protocols were equally effective for circumferential and focal ablation [32,33].

However, as the simplified regimen for focal RFA (3 × 15 J/cm^2^, without cleaning) resulted in a higher stenosis rate, a new simplified protocol with lower energy (3 × 12 J/cm^2^, without cleaning) was developed. A multicentre randomized trial showed the non-inferiority of the simplified protocol in terms of safety and efficacy compared to the standard protocol, significantly shortening the average procedure time of focal RFA [34]. 

In circumferential ablation, radiofrequency energy is delivered uniformly through the electrodes at a preset energy density of usually 12 J/cm^2^, ablating a depth of 700–1000 µm over 3 cm area. Although novel self-sizing ablation balloon catheters, such as the Barrx 360 Express, allow one to avoid a separate measuring procedure, the older conventional ablation catheters still need the measurement before its introduction. For this purpose, the scope is removed and replaced with a guidewire. The endoscopist measures the oesophagus diameter in a stepwise manner by introducing a sizing balloon over the guidewire and starting to take measurements from 6 cm above the BE proximal extension down until the balloon reaches the stomach. The sizing balloon is then removed, and depending on measurements, the endoscopist select the appropriately sized ablation catheter [31].

After this separate measuring procedure, the ablation catheter is advanced over the guidewire followed by the endoscope and it is positioned 1 cm above the proximal end of the BE segment. Then, the ablation catheter is inflated and radiofrequency energy is delivered through the device to the apposed BE segment. After that, the ablated mucosa is cleaned by removing the debris and coagulum and RFA is then reapplied. The ablation catheter is then advanced down the BE segment and the process is repeated, carefully avoiding > 1 cm of overlap, until RFA has been applied twice overall.

The traditional system was recently substituted by the 360 Express RFA balloon catheter, which can self-adjust to the oesophageal lumen providing an excellent apposition during each ablation. While circumferential RFA with 360 Express RFA balloon catheters showed that it is equally effective and less time-consuming than the older system, the increased tissue contact may lead to a higher depth of penetration, increasing the risk of stenosis [35]. Thus, the energy density settings for the 360 Express RFA balloon catheter were lowered from 12 J/cm^2^ to 10 J/cm^2^ [35]. A multicentre randomized clinical trial demonstrated that, when using 360 Express RFA balloon catheters, the standard regimen (1 × 10 J/cm^2^-clean-1 × 10 J/cm^2^) should be preferred to the simplified regimen (2 × 10 J/cm^2^, without cleaning), as the latter was associated with a relevant risk of stricture [36]. Thus, the simplified regimen is indicated for focal RFA and circumferential RFA with conventional devices, while the standard protocol is recommended when using novel circumferential ablation devices.

Overall, as its efficacy and safety is supported by robust, long-term evidence [12,37,38,39,40,41,42], RFA is currently regarded as the first-line ablation technique for the treatment of flat dysplastic BE (Table 2).

Nevertheless, RFA, although well-established and -validated, may not always be effective at leading to CE-D and CE-IM. In addition, it is also expensive and not available in many countries and, thus, alternative options are certainly needed. These other ablative techniques are less well-validated but have shown encouraging results. Moreover, the presence of strictures or uneven BE surface, impairing tissue adherence, may lead to an ineffective RFA; other techniques, such as cryospray are preferred in these cases.

### 4.2. Argon Plasma Coagulation

APC uses a contact-free probe, passed through the endoscope, to deliver energy applying ionized argon gas to the target tissue, leading to thermal destruction. APC is usually applied with energy settings ranging from 30 to 90 W at a rate of 1–2 L/min. Due to its simplicity, APC was one of the most used techniques for the ablative treatment of BE in the old days.

It has been shown to be an effective ablative option (CE-IM rates of 58–78%) and to lower the risk of progression to HGD and EAC when compared with surveillance alone [43,44]. Moreover, despite the poor homogeneity of data concerning its efficacy and durability in obtaining CE-D, APC has demonstrated to achieve high initial CE-D rates (more than 95%) in BE with LGD [45]. 

However, the APC technique does not ensure a homogenous application of thermal energy over the whole BE segment. On the contrary, the energy delivered tends to be higher at the initial site of treatment and lower as the probe is moved along the BE tract. The exaggerated amount of energy conveyed to the initial site can lead to potential deep tissue injury, whereas a scarce delivery can result in an unsuccessful treatment with persistence of buried BE glands under the neo-squamous epithelium [46]. Indeed, a high rate of recurrence of both metaplasia and dysplasia in more than one third of treated patients was reported at long-term follow-up with a 3% annual risk of developing EAC, which is comparable to the risk of those BE patients who did not undergo ablative therapy [47].

Furthermore, 10% of patients treated with APC suffered from severe adverse events, including bleeding, strictures and perforation because of the large depth of tissue injury; notably, the risk of developing post-treatment strictures was between 4% and 9% [44].

Thus, APC has fallen into disuse due to these remarkable limitations in favour of newer and safer ablative options.

**Figure 2:**
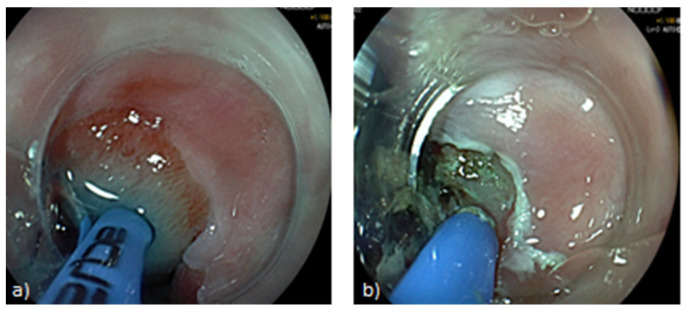
Treatment of Barrett’s oesophagus with hybrid argon plasma coagulation. (**a**) Submucosal injection of normal saline through the APC probe. (**b**) Ionized argon gas is delivered, leading to thermal destruction of target tissue.

Recently, Manner et al. described a modified technique, called “Hybrid-APC”, as an alternative ablation method with lower risk of deep tissue damage. It entails the combination of conventional APC with prior submucosal injection of normal saline, through a fluid jet channel built into the APC probe (Figure 2) [48]. A randomized controlled trial, enrolling 50 patients with BE who underwent hybrid APC, showed CE-IM was achieved by 78% of patients after a median of 3.5 ablative sessions, while only 2% developed strictures [13]. These data made hybrid APC a promising novel ablating technique, opening the road for further investigations. A pilot study, recruiting 22 patients, confirmed high rates of CE-IM (86.4%) with an acceptable safety profile (two cases of strictures) [49]. Very recently, a multicentre prospective trial by Knabe et al. assessed efficacy and safety of hybrid APC as a therapy for BE-related neoplasia. A total of 148 patients underwent hybrid APC after endoscopic resection or as a primary treatment. After a mean of 2.7 ablation sessions, 98% and 88.4% of patients reached complete eradication of neoplasia and CE-IM, respectively. Among those successfully treated, 65.9% showed sustained CE-IM at 2 years follow-up. A complication rate of 6% was reported, mostly including strictures (3.9%) which were successfully treated with dilation [50].

Additionally, a small meta-analysis and systematic review, including 202 patients from six studies, was carried out to assess the efficacy and adverse events rate of hybrid APC. Overall, CE-IM was achieved by 89.6% of patients in a mean number of treatment sessions ranging from 1.2 to 2.7. Major complications occurred in 2.5% of cases, with an overall stricture rate of 1.3% [51].

Furthermore, a small retrospective cohort study was performed to compare safety and CE-IM rates of hybrid APC versus RFA. A total of 27 patients who were treated with hybrid APC were matched against 27 other patients who underwent RFA according to baseline features (e.g., gender, age, histology, length of BE etc.). CE-IM was achieved in 74% of the patients in the RFA group and in 89% of the subjects in the hybrid APC group; however, statistical significance was not reached (*p* = 0.16). Regarding safety, while 44% of patients treated with RFA developed a post-treatment adverse event, including 4 cases of stricture (15%), no complication occurred in the hybrid APC group (*p* < 0.0001) [52].

These results show hybrid APC is a feasible, safe and effective ablative technique. It is currently regarded as a second-line therapy after the failure of RFA. However, despite the evidence still being limited, these findings suggest hybrid APC is at least equally effective as focal RFA in the treatment of BE, with an even better safety profile, paving the way for randomized trials comparing these options as first-line ablative treatment for BE. In addition, given the significant price difference between hybrid APC and RFA, this modality may be more cost-effective. Two multicentre randomized controlled clinical trials comparing hybrid APC vs. RFA are currently ongoing to further understand the efficacy and safety of this technique in comparison to RFA [53,54]. 

### 4.3. Cryotherapy

Cryotherapy is an ablative therapy, which, through repeated cycles of rapid freezing and slow thawing of diseased mucosa, leads to thermal injury and necrosis of metaplastic tissue. The fast-freezing phase generates intra- and extra-cellular ice crystals, which induce apoptosis, damaging cell membranes. During the thawing phase, further injury is caused to cell membranes, such as thrombosis of local blood vessels. After the application of cryotherapy, the treated epithelium typically develops a cherry red appearance [55].

Cryotherapy encompasses two techniques. Cryospray with liquid nitrogen (the TruFreeze system) or carbon dioxide (Polar Wand) as cryogenic fluid is the older non-contact ablative approach. First, an orogastric decompression tube is advanced over a guidewire into the gastric cavity to vent the stomach to prevent perforation due to excessive gastric distension caused by nitrogen gas that evaporates during the procedure. Then, the endoscope is advanced alongside the tube and the cryotherapy catheter is passed through the scope and positioned between 0.5 and 1 cm from the diseased epithelium. At this point, spray is delivered at about −196 °C (the TruFreeze system) or −80 °C (Polar Wand) to a hemi-circumferential portion of BE segment and the site is frozen for 20–30 s, followed by thawing for at least 45–60 s. To achieve an appropriate depth of ablation, three freeze–thaw cycles are usually performed, even if there is lack of standardization regarding the time duration of each phase and the number of cycles. Then, process is repeated until spray cryotherapy has been delivered to all the metaplastic tissue [55]. Notably, cryospray is contraindicated in case of altered gastric anatomy, such as in gastric bypass, stomach stapling and gastrojejunostomy due to the increased risk of perforation.

**Figure 3:**
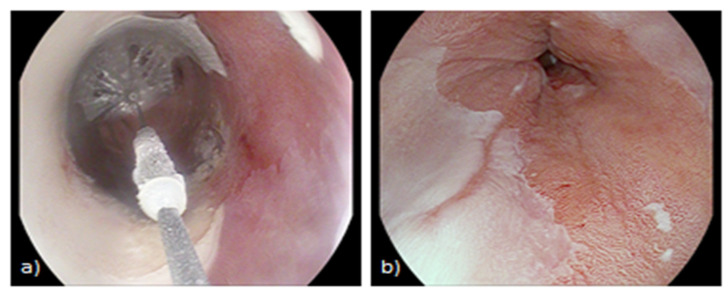
Application of the CryoBalloon focal ablation on a Barrett’s segment. (**a**) The cryogen is delivered perpendicularly to the balloon wall through a 1 mm opening within the balloon. (**b**) Cherry red appearance of mucosa after treatment.

The other newer cryotherapy modality is the CryoBalloon Focal Ablation System (CbFAS), which uses nitrous oxide as cryogen and a cryotherapy catheter balloon to ablate target tissue. It is a portable device that is self-venting since nitrous oxide is contained within the balloon that contacts the targeted site. The cryotherapy catheter balloon, attached to a handheld trigger, is passed through the scope and advanced to the target area. As the balloon is inflated by the trigger, it can self-adjust its size according to oesophagus’ diameter, and the cryogen is delivered through the continued activation of the trigger through a 1 mm opening within the balloon itself, directed perpendicularly to the balloon wall. This turns into focal cryoablation of the mucosa alongside the balloon, usually to −85 °C for 10 s per site. The site of delivery can be accurately controlled through the rotation and advancement of catheter using the foot pedals of the CryoBalloon ablation system [55]. Thus, CbFAS has some advantages over spray cryotherapy. First, it is portable and takes little space. Furthermore, as the evaporated gas is expelled from within the balloon, a decompression tube is not needed. Additionally, since cryogen is contained within the balloon instead of being released into the lumen, repeated freeze–thaw cycles are not required, and freezing is more efficient and quicker with an average procedural time of only 11 min. Moreover, whilst cryogen spray can affect visualization, the focal ice patches and the erythematous mucosal change are well-visible with CbFAS and targeted ablation can be thus accurately performed, reducing the risk of treatment overlap and ablation of normal mucosa (Figure 3). In addition, while treatment of residual areas within strictures can be difficult with other options, the CbFAS allows a more stable position to treat these difficult sites minimizing risk of perforation. This benefit has been further enhanced by the recent introduction of next-generation CryoBalloon devices, encompassing the availability of a pear-shaped balloon to better ease adherence at the GEJ, allowing a circumferential ablation of this site, and wider probes to ablate larger areas [56]. Lastly, focal cryoballoon ablation is less expensive and may be more cost-effective than other ablative techniques.

Although comparative randomized trials between ablative techniques are lacking, recent studies have demonstrated that cryotherapy, either in the form of cryospray or cryoballoon forms, has achieved encouraging results in terms of efficacy and safety profile.

A multicentre retrospective cohort study, recruiting 98 patients with BE-HGD, reported liquid nitrogen-based spray cryotherapy achieved CE-D and CE-IM in 87% and 57% of patients, respectively. Additionally, a good safety profile was observed with a stricture rate of 3%, while post-procedural chest pain occurred in only 2% of patients. Despite being encouraging, these results were hugely limited due to the short follow-up duration and the study design [57].

Later, a single centre retrospective study with long-term follow-up, enrolling 50 patients with BE-HGD or EAC, reported liquid nitrogen-based spray cryotherapy achieved CE-D and CE-IM in 88% and 75% of patients, respectively, at 5 years of follow-up. Recurrence rates of BE, LGD, and HGD/EAC after achieving CE-IM were 12.2%, 4.0%, and 1.4%, respectively, per person-year of follow-up. Only 2 HGD patients (4%) developed EAC despite therapy [15].

A recent systematic review and meta-analysis evaluated the safety and efficacy of liquid nitrogen-based spray cryotherapy as a treatment of BE, including 386 patients from nine studies. The overall pooled rates of CE-IM and CE-D were 56.5% and 83.5%, respectively. Notably, pooled rates of CE-IM and CE-D were 58.4% and 81.9% among patients who had previously failed RFA, whereas CE-IM was achieved by 53.7% of treatment-naïve patients. BE recurrence occurred in 12.7% of cases, while only 4.7% suffered from complications, the most common of which were stricture and chest pain [58].

In the first prospective clinical trial assessing safety and efficacy of the CbFAS on 41 patients with BE-dysplasia or intramucosal cancer (IMC), CE-D and CE-IM rates at 1 year were 95% and 88%, respectively; the CE-D rate was significantly higher (100%) in patients with BE < 8 cm compared to those with BE ≥ 8 cm (67%, *p* = 0.02). A median of three sessions was required. Post-treatment strictures developed in 9.7% of patients and needed endoscopic dilation [14].

Later, CbFAS was evaluated through a systematic review and meta-analysis carried out by Westerveld et al., including seven trials with a total of 272 patients with BE dysplasia/early EAC. CbFAS demonstrated a high rate of technical feasibility (95.8%). The overall pooled rates of CE-D and CE-IM were 93.8% and 85.8%, respectively. The pooled complication rate was 12.5%, with a stricture development rate of 5.8%. However, these results were hugely limited due to the very short follow-up period and potential high degree of heterogeneity as both treatment naïve and RFA non-responders were enrolled [59].

A subsequent prospective single-arm multicentre study assessed the efficacy and safety profile of CbFAS as a first-line treatment for BE-related dysplasia/early EAC. In this study, 120 treatment naïve patients with BE of 1–6 cm length were recruited and underwent nitrous oxide-based cryoballoon focal ablation for all visible BE epithelium up to five sessions. At 1-year follow-up, with the intention to treat analysis, CE-D and CE-IM was achieved in 72% and 76% of patients, respectively, whereas, in the per-protocol analysis, 97% and 91% of patients achieved CE-D and CE-IM, respectively. Post-ablation strictures occurred in 12.5% of cases [60].

To optimize the dosing and protocol of focal cryoballoon ablation, a recent prospective multicentre study, enrolling 56 patients with BE dysplasia, compared the outcomes of 8 s cryoballoon ablation versus the standard 10 s. After a single ablation session, the median BE surface regression did not differ between the two groups (80%, *p* = 0.65). The stricture development rates were also comparable between the 8 s and the 10 s group (15 vs. 19%, *p* = 1.00). Two patients of the 10 s group had severe strictures, which required more than three dilations. Therefore, as the efficacy outcomes seem not to be affected by lowering the ablation dose, 8 s cryoballoon ablation may be regarded as the preferred regimen in order to decrease the risk and severity of strictures. A large, multicentre, prospective study assessing the efficacy and safety of CbFAS for treatment naïve patients with dysplastic BE is currently ongoing (Coldplay III) [61]. 

Since RFA has been established as the first-line ablative option for BE due to its well-documented safety and efficacy, cryotherapy has mostly been regarded as a rescue therapy after the failure of RFA and is currently considered a second-line option for refractory disease.

A systematic review and meta-analysis carried out by Visrodia et al., including 11 studies with 148 patients with persistent IM or dysplasia after RFA, showed cryotherapy (either liquid nitrogen- or carbon dioxide-based cryospray, or nitrous oxide-based CbFAS) as a second-line therapy after RFA failure, achieved a CE-IM and CE-D of 45.9% and 76%, respectively. A good safety profile was reported (adverse events in 6.7% of patients) [62]. 

Furthermore, given these encouraging results, researchers have investigated the potential role of cryotherapy as primary therapy for BE-related neoplasia [63,64,65,66,67] (Table 3). 

Overall, liquid nitrogen spray cryotherapy is a feasible, safe and effective ablative technique, which may become an alternative modality to RFA as first-line option for dysplastic BE. Moreover, although available data are currently limited and a negligible stricture rate has been reported, cryoballoon ablation is a promising, valuable and relatively low-cost ablative option, which may enhance technical feasibility and decrease procedural time. Nevertheless, randomized high-quality comparative trials between RFA and cryotherapy techniques are eagerly awaited.

## 5. Post-Procedural Care and Surveillance

After ablative procedures, patients may experience chest pain, which should be controlled with tramadol or acetaminophen. Patients are usually able to be discharged to go home, some hours after the procedure, without requiring hospitalization. They should be kept on a liquid diet for approximately 24 h post-procedure; then, they can resume their habitual diet as tolerated. Beyond PPI, which should be continued in the long run, typical maintenance therapy after procedure encompasses sucralfate in the immediate post-procedural period.

Since the pooled incidence rate of BE recurrence ranged from 8.6% to 10.5%, and the incidence of dysplastic and early EAC recurrences were 2.0% and 1.2%, respectively, according to a meta-analysis, taking into account all endoscopic treatment options, close surveillance is mandatory [48]. Until some years ago, surveillance endoscopies for patients with baseline HGD or IMC, after achieving CE-IM, were scheduled every 3 months for the first year, every 6 months for the second year, and annually afterwards; for those with baseline LGD, after achieving CE-IM, endoscopy was recommended every 6 months in the first year and annually afterwards [68].

However, a recent study conducted by Cotton et al., based on the recurrence rate from the US RFA patient registry and UK National Halo Registry, showed a dysplasia recurrence risk of 2.9% with a subsequent EAC risk of 0.1%, which was used as a threshold to develop novel wider surveillance intervals [69]. As this adjustment led to a significant decrease in the number of surveillance endoscopies and related costs, without affecting detection of neoplastic recurrence, longer surveillance intervals are recommended by the latest updated guidelines. Thereby, surveillance endoscopy in patients with baseline HGD or IMC, after achieving CE-IM, is currently recommended every 3 months for the first year and yearly afterwards. In patients with baseline LGD, after achieving CE-IM, surveillance endoscopy is recommended at 1 and 3 years, and every 2 years thereafter [7,19].

Surveillance endoscopy should include a meticulous assessment with high-resolution white light endoscopy and virtual chromoendoscopy of the neosquamous epithelium and GEJ looking for any visible lesions. Random 4-quadrant biopsies should be taken from the distal 2–5 cm of the neosquamous epithelium and the GEJ. If IM, dysplasia or sub-squamous columnar epithelium (namely, buried BE) is detected on random biopsies from the neosquamous epithelium, additional ablation therapy should be applied, whereas the isolated detection of IM from the gastric cardia should not be treated [7,19].

## 6. Conclusions

In the last two decades, the therapeutic approach for BE-related dysplasia and early EAC has completely changed, shifting from esophagectomy to EET, which has been established as the gold standard due to excellent efficacy and safety outcomes. While there is a large body of evidence supporting RFA as first-line choice among ablative options, newer technologies, such as hybrid APC and cryotherapy techniques, are being increasingly refined and investigated as novel ablative therapies for BE. Based on the available literature, hybrid APC and cryotherapy have achieved very promising results in terms of feasibility, efficacy, safety and cost-effectiveness, which make them appealing not only as rescue-therapies after RFA failure, but even as potential alternative first-line treatments. Given these encouraging findings, large, randomized controlled trials comparing ablative techniques are ongoing and many others will hopefully be conducted in the near future to elucidate which technique should be preferred. However, while awaiting further evidence, at the moment, RFA should still be regarded as the gold standard therapy for ablation of BE dysplasia and early EAC.

## Figures and Tables

**Table 1:** Focal radiofrequency ablation applied through Barrx 90 RFA focal catheter.

Principal Devices for RFA Ablation	Focal/Circumferential	Company	Electrode Dimension and Ablation Are	Use Modality
The Barrx 360 Express RFA balloon catheter	Circumferential	Medtronic Inc., Sunnyvale, CA, USA	From 18 to 31 mm Ablation over a distance of 4 cm	over-the-wire
Barrx 90 RFA focal catheter	Focal	Medtronic Inc., Sunnyvale, CA, USA	20 mm (l) × 13 mm (w) (ablation area 2.6 cm^2^)	over-the-scope
Barrx Ultra Long RFA focal catheter	Focal	Medtronic Inc., Sunnyvale, CA, USA	40 mm (l) × 13 mm (w) (ablation area 5.2 cm^2^)	over-the-scope
Barrx 60 RFA focal catheter	Focal	Medtronic Inc., Sunnyvale, CA, USA	15 mm (l) × 10 mm (w) (ablation area 1.6 cm^2^)	over-the-scope
The Barrx Channel RFA endoscopic catheter	Focal	Medtronic Inc., Sunnyvale, CA, USA	7.5 mm × 15.7 mm distal electrode (ablation area 1.2 cm^2^)	through-the-scope

**Table 2:** Main articles on radiofrequency ablation technique: BE: Barrett oesophagus, CE-IM: complete eradication of intestinal metaplasia, CE-D complete eradication of dysplasia, RFA: radiofrequency ablation, LGD: low grade dysplasia, HGD: high grade dysplasia, EAC: oesophageal adenocarcinoma, EET: endoscopic, endoscopic eradication therapy.

Authors	Field	Study Features	Main Findings
Shaneen et al. [22] The AIM study	RFA rates of eradication procedure	Multicentre Randomized sham controlled-trial, 127 patients. Primary outcome: CE-IM and CE-D one year	Dysplasia eradication rate 98% at one year after the RFA procedure against 22% of sham procedure (*p* < 0.001)
Cotton [41]	RFA outcome	Long term rate of eradication and recurrence rate of the RFA in the cohorts of the AIM study	Incidence rate of BE recurrence was 10.8 per 100 person-years overall. Greater probability of recurrence in the first year following CEIM than in the following 4 years combined
Phoa [12] The SURF study	RFA ablation vs. surveillance at 5 years	Multicentre randomize trial, RFA ablation vs. endoscopic surveillance Primary outcome neoplastic progression to HGD or EAC during a 3-year follow-up.	RFA reduced the risk of progression to HGD or EAC by 25.0% and the risk of progression to EAC by 7.4%
Pouw [37]	RFA long term outcome	Same cohort of the SURF with additional 40 months of follow-up	RFA of BE with confirmed LGD significantly reduces the risk of malignant progression, with sustained clearance of BE in 91% and LGD in 96% of patients, after a median follow-up of 73 months
Wang [25]	RFA vs. endoscopic surveillance	Progression BE-LGD to HGD and/or EAC after treatment with RFA and endoscopic surveillance.	Pooled estimate of rate of neoplastic progression of BE-LGD to HGD or EAC was much lower in the RFA group than the endoscopic surveillance group) RFA decreases the risk of BE-LGD progression to BE-HGD.
Barret [42]	RFA vs. Surveillance in LGD	82 with confirmed LGD randomised, 40 patients in the RFA and 42 in the surveillance group. Primary outcome: prevalence of LGD at 3 years	in the surveillance group (OR = 0.38 (95% CI 0.14 to 1.02), *p* = 0.05) RFA modestly reduced the prevalence of LGD as well as progression risk at 3 years.
Krishnamoorthi [38]	RFA treatments vs endoscopic surveillance	21 RFA studies that reported recurrence in 603/3186 patients, with over 5741 patient-years of follow-up	Pooled overall incidence rates of recurrent BE, LGD and HGD/EAC after RFA were 9.5%, 2% and 1.2% per patient-year.
Komanduri [39]	PPI therapy post RFA therapy	BE patients referred for EET managed with a standardized reflux management including twice-daily PPI, therapy during eradication Primary outcomes rates of CE-IM and IM or dysplasia recurrence	Importance of reflux control in patients with BE undergoing EET.
Qumseya [40]	RFA vs. EMR ablation technique	37 article (9200 patients RFA showed 5.6%, strictures, 1% bleeding, 0.6% perforation.	RFA to be about 4-fold higher with EMR than without oesophagus and length and baseline histology as risk factors for adverse event.

**Table 3:** Main articles about Cryotherapy ablation technique: CE-IM: complete eradication of intestinal metaplasia, CE-D complete eradication of dysplasia, RFA radiofrequency ablation.

Authors	Field	Study Features	Main Findings
Hamade [63]	Cryotherapy as first-line treatment	6 studies, 282 patients, 459 person years of follow-up.	CE-IM rate: 69.35% CE-IM, CE-D: 97.9%. Neoplasia recurrence rate 10.4%.
Tariq [64]	Cryotherapy as first-line therapy	Meta-analysis including 405 patients with follow-up ranging from 3–54 months.	Cryotherapy CE-D reached a pooled proportion of 84.8%.
Thota [65]	RFA and cryotherapy comparison	154 patients included, 73 patients were in the RFA and 81 patients were in the cryotherapy group.	Cryotherapy is similar to RFA in CE-D endpoint (but inferior in CE-IM.
Fasullo [66]	RFA vs. liquid spray cryotherapy	100 patients in the RFA group and 62 patients in the liquid spray cryotherapy group.	Cryotherapy is similar to RFA in CE-D and CE-IM but require more session.
Agarwal [67]	RFA vs. cryoballoon cohort study	Propensity score-matched analysis in a cohort study, RFA vs. cryobaloon ablation.	Comparable chance of achieving CE-IM. Cryoballoon group had a higher stricture rate compared to RFA.

## Data Availability

No new data were created.
